# Application of the Hurdle Technology Concept to the Fresh Za’atar (*Origanum syriacum*) Preservation

**DOI:** 10.3390/foods11193002

**Published:** 2022-09-27

**Authors:** Samer Mudalal, Doaa Kanan, Ola Anabtawi, Alma Irshaid, Mohammed Sabbah, Munqez Shtaya, Faisal Shraim, Gianluigi Mauriello

**Affiliations:** 1 Department of Nutrition and Food Technology, Faculty of Agriculture and Veterinary Medicine, An-Najah National University, Nablus P.O. Box 707, Palestine; 2 Department of Plant Production and Protection, Faculty of Agriculture and Veterinary Medicine, An-Najah National University, Nablus P.O. Box 707, Palestine; 3 Department of Agricultural Science, University of Naples Federico II, 80055 Portici, Italy

**Keywords:** oregano, vacuum packaging, *Allium cepa*, *Rhus coriaria*, refrigeration

## Abstract

Oregano (*Origanum syriacum*) is popularly called *za’atar* in the Middle East region. It is widely used in the Mediterranean diet as an aromatic herb. This study aimed to evaluate the preservation effect of natural additives, vacuum packaging, and refrigeration on the quality traits of fresh oregano. In total, 132 fresh oregano samples were formulated and split into 4 groups (*n* = 33) labeled group A (100% fresh oregano leaves, Control), group B (fresh oregano 63.2%, 15% fresh onion, 20% oil, 1.8% salt), group C (fresh oregano 61.91%, 15% fresh *Allium cepa*, 20% oil, 1.8% salt, 1.29% sumac), and group D (fresh oregano 59.2%, 15% fresh *Allium cepa*, 20% corn oil, 1.8% salt, 4% lactic acid, ultimate pH 4.4). Different quality traits such as color index (L*a*b*), microbiological analysis (total aerobic, anaerobic, and psychrotrophic bacteria and yeasts and molds), and sensory features (taste, flavor, appearance, saltiness, and overall acceptance) were assessed during the storage period (42 days) for all groups. Our study showed that the addition of lactic acid (group D) exhibited a strong preservation effect against aerobic and anaerobic bacteria. In this context, group D had significantly lower aerobic and anaerobic bacterial counts (5.12 vs. 6.7, 6, and 6.7 log (cfu/g); *p* < 0.05) and (4.75 vs. 6.6, 6.1, 6.77 (cfu/g); *p* < 0.05) than group A, B, and C; respectively. Group D exhibited significantly (*p* < 0.05) lower psychrotrophic bacterial count (3.6 log (cfu/g)) during the whole period of storage compared with control. Group B had a lower redness index (a*) (−3.3 vs. −1.8, −1.65, −1.23; *p* < 0.05) than groups A, C, and D; respectively. In conclusion, our study showed that there is a possibility of improving the preservation of oregano (*Origanum syriacum*) by using lactic acid and sumac combined with vacuum packaging under refrigeration conditions.

## 1. Introduction

*Origanum* species are widely grown and cultivated in the Mediterranean basin. Out of the 43 species, 35 species occur solely in the East Mediterranean [1,2]. *Za’atar* is one the most common names for *Origanum syriacum* [3], and it is frequently used for different purposes and in different forms in Mediterranean cuisine, due to its fragrance, flavor, and properties that it adds to food, such as meat, vegetables, and baked goods [4].

Oregano has been used in the past to formulate a traditional medicine for several diseases. In this context, the traditional medicine containing oregano was dedicated to treating respiratory diseases such as whooping cough, bronchitis, and asthma [5]. The most common functional ingredients in oregano leaves are thymol and carvacrol [6]. *Origanum syriacum* leaves are considered a rich source of vitamins such as folic acid, beta carotene, and vitamins E, K, A, and C [7,8]. Its leaves are rich in phenolic compounds such as caffeic acid, rosmarinic acid, eriodyctiol, luteolin, naringenin, and apigenin [9,10,11]. Spain, France, Italy, Switzerland, Bulgaria, Portugal, and Greece among other European countries cultivate and harvest wild thyme and oregano [12].

The oregano crop (*Oregano syriacum*) is a major component of the agricultural economy in Palestine; it is also called Palestine’s green gold. Palestinians have known different types of oregano, especially wild oregano, and used it in their traditional cuisine. A few years ago, Palestinians realized how much its cultivation is due to its economic feasibility [13].

Thymol is one of the components of the essential oil of oregano that has antiseptic and antifungal properties [14,15]. Moreover, oregano has other volatile oils such as carvacrol, geraniol, and borneol that have antimicrobial properties. In addition, thyme contains linalool, apigenin, eugenol, and rosmarinic acid, which have antioxidant, anti-inflammatory and antiviral properties [10,11,14,15,16]. The studies revealed that thyme and oregano contained a significant amount of phenolic compounds such as zea-xanthin, which acts as a bronchodilator; apigenin, a muscle relaxer; and lutein, which supports brain and vision development, as well aso luteolin and thymosin [5,16]. The diversity of geography and climate in Palestine contributes to farm-wide varieties of *Origanum syriacum* species including wild and cultivated [17]. Several researchers investigated the ability to preserve *Origanum syriacum* by vacuum packaging [18] or by solar and freeze drying [19]. Mudalal and Abu-Khalaf [20] found a significant difference in some quality traits between freeze- and solar-dried *Origanum syriacum* using an electronic nose.

The market for *Origanum syriacum* has different channels for rural families and small farmers who can sell to local trades or small stores. In addition, some companies have private labels. There are numerous challenges that limit the exporting of *Origanum syriacum* including restricted and complicated access to fertile land (in particular in area C: political classification by the Israeli occupation); lack of quality control on agricultural inputs and practices; limited water for irrigation; and weak cold chain (for fresh and frozen products), which result in high waste during transit. On the other hand, dried *Origanum syriacum* exports have rapidly increased because of high demand in the region.

Oregano is only marketed as fresh in winter and spring, as it exhibits an unacceptable bitter taste in the remaining seasons. To overcome shortages of production due to off-seasons, our local small industries employ several conventional preservation techniques such as solar-drying, freezing, and packaging in plastic bottles. Similar techniques are usually used in other Mediterranean regions. However, the current conventional preservation techniques have adverse effects on the nutritional value and sensory characteristics (mainly color, taste, and flavor). So far, current conventional preservation techniques are not able to maintain the freshness of oregano, which is a very important issue for the production of local oregano-based bakery products. In the Mediterranean region and particularly in Palestine, fresh oregano leaves are usually mixed with oil, fresh onion, sumac, and salt then stuffed in flour dough in the form of a thin sheet, followed by baking. Our research aimed to study the preservation effects of refrigeration storage, vacuum packaging, and natural ingredients on the quality traits of fresh oregano.

## 2. Materials and Methods

### 2.1. Collection and Preparation of Samples

A small oregano field was cultivated in a village near Tulkarem city (Northern Palestine) and was used to collect about 5 kg of oregano stems. The selected areas in the field for sample collection had no weeds or plant diseases. The leaves were removed from the stems by hand. The final net weight of separated leaves after the removal of straw, stems, gravel, and any type of physical impurities was about 2.5 kg. Ultimately, the oregano leaves were subjected to a cleaning process to remove any types of soils and dust using running tap water. The cleaning process was stopped when the output water became clear. The wetted leaves were left to dry at room temperature for about one hour over paper towels to remove excess water due to the washing process, and the drying process did not remove any part of the native water in the fresh leaves. The whole quantities of leaves were mixed thoroughly to obtain a homogenous mixture. The total quantity of oregano was split into four batches to represent four treatments. In each group, there were 33 packs. Each pack contains a net weight of about 60 g of the product. Accordingly, the total number of samples for all treatments was 132.

**Figure 1:**
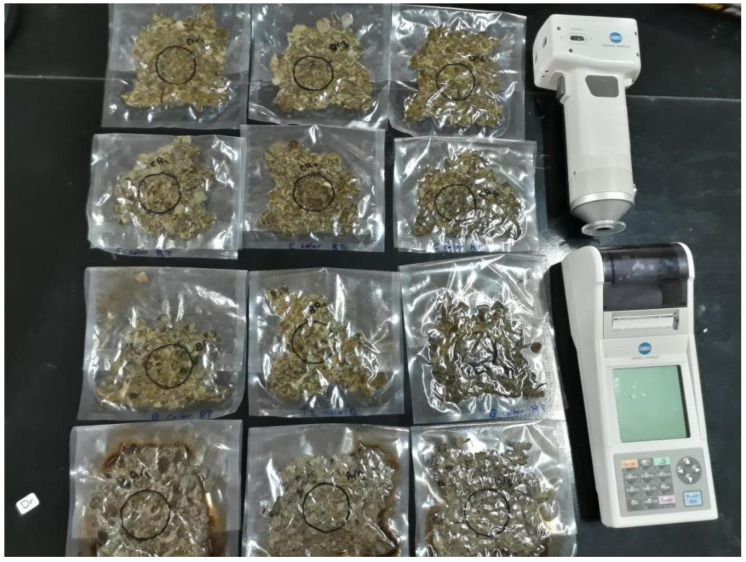
Oregano mix as the finished product was prepared for color index (L*a*b*) measurement.

In each group, different natural ingredients were added to oregano leaves, and then the ingredients were mixed and packaged under vacuum (−95% ambient pressure for 10 s, Figure 1). Transparent vacuum plastic packs made of coextruded multilayer flexible film with dimensions of 200×300 mm. The groups were as follows:

Group A: (Control group): (100% Fresh oregano leaves)

Group B: (73.2% Fresh oregano, 15% Fresh *Allium cepa*, 20% corn oil, 1.8% salt)

Group C: (61.9% Fresh oregano, 15% Fresh *Allium cepa*, 20% corn oil, 1.8% salt, 1.29% sumac “*Rhus coriaria*”)

Group D: (59.2% Fresh oregano, 15% Fresh *Allium cepa*, 20% corn oil, 1.8% salt, 4% lactic Acid, ultimate pH 4.4).

The percentages of ingredients were calculated by weight.

The samples were stored under refrigeration conditions (2–4 °C), and the relative humidity was about 40–60%.

### 2.2. Chemical Analysis

About 100 g of fresh oregano leaves was dedicated for proximate chemical analysis (moisture, fat, protein, fiber, and ash content). The proximate chemical composition of fresh oregano was assessed for each sample using official methods of AOAC [21]. Moisture content was determined by loss on drying using an air oven. About 5 g of the sample was accurately weighed and then dried at 105 °C for 16 h. The moisture content was calculated based on weight differences. For ash, about 2 g of fresh leaves of oregano was accurately weighed in the crucibles and incinerated in the muffle furnace for 5 h at 550 °C. After incineration, the weight of ash was recorded, and then ash content was calculated. The fat content was determined using an ANKOM XT15 extractor. About 1.5 g of fresh leaves of oregano was placed in the filter bags, then the fat extracted by petroleum ether in the extraction vessel. The fat content was calculated based on weight differences (before and after extraction). Fiber content was measured using an ANKOM 200 Fiber Analyzer. About 0.5 g of dried oregano leaves was placed in the filter bag. After that, the filter bags were placed in a fiber analyzer using the suspender tray. The samples were first digested using a heated acid solution (90 °C), followed by water washing and digestion using a heated alkali solution (sodium hydroxide). The remaining undigested matter was considered as total crude fiber.

### 2.3. Color Measurement

The CIE system (Commission Internationale de l’Eclairage) was used to measure color parameters according to standard values that are internationally used.

In each group, six different areas were highlighted with black circles over each pack. Color coordinates were measured in triplicates for each area, and the mean was determined. A reflectance colorimeter (Minolta Chroma Meter CR-400) with an illuminant source was used to measure the color index (L*a*b*). A reference white ceramic tile (Y = 93.9, x = 0.3130 and y = 0.3190) was used to calibrate the colorimeter before each measurement.

### 2.4. pH Measurement

Ten samples were selected from each group for pH measuring. About 2.5 g of fresh oregano was added to 25 mL of distilled water and then homogenized with ultra-turrax. The pH meter (ISFET, Model 98 # IQ150, IQ Scientific Instruments, San Diego, CA, USA) was calibrated at pH 4.0 and 7.0 before measuring the samples.

### 2.5. Microbiological Analysis

#### 2.5.1. Aerobic, Anaerobic, and Psychrotrophic Bacterial Count

Total aerobic, anaerobic, and psychrotrophic bacteria counts were estimated in 12 replicates during the period of the study (42 days). 10 g of oregano samples was aseptically added with 90 mL ringer solution. Seven dilutions (10^−1^ to 10^−7^) were used to count bacteria, mold, and yeast. Plate Count Agar (PCA) was used as a microbiological growth medium to estimate the total viable bacterial count. The plates were incubated for 48–72 h at 37 °C. Similar incubation conditions were used to count anaerobic bacteria, but the plates were kept in an anaerobic jar. Plate count agar (PCA) was also used to count psychrotrophic bacteria, and the plates were stored in a refrigerator for 7 days. The plates containing 25–250 colonies were considered for counting.

#### 2.5.2. Yeast and Mold

From each group, four different samples were selected to determine yeast and mold counts. Potato dextrose agar (PDA) was used as a culture medium. Serial dilutions (10^−1^ to 10^−5^) were used to obtain proper colony counts. The plates were incubated for 4–5 days at room temperature. The plates containing 25–250 colonies were considered for counting.

### 2.6. Sensory Analysis

Three packs of oregano samples were selected from each group to prepare bread stuffed with oregano mix as normally prepared in local bakeries that are already present in Palestine. The dough for the bread was made from wheat flour, dried yeast, salt, and a little sugar to activate the yeast. The dough was rolled into a thin layer, then stuffed with oregano mix and baked at 250 °C until the formation of a gold crust. Bread with oregano (*qraas za’atar*) was cut into small similar pieces. These pieces were subjected to sensory analysis by 30 panelists (trained) to evaluate 5 descriptors, taste, flavor, appearance, saltiness, and overall acceptance. The panelists were trained before they started the sensory analysis by explaining the scale hedonic test (in Arabic). The panelists expressed the intensity of each attribute with a mark on a 9-point scale (9 = Like Extremely; 1 = Dislike Extremely). The samples were coded in randomized blocks and presented to the panelists on plastic plates.

### 2.7. Statistical Analysis

Each quality trait (proximate analysis, color traits (L*a*b*), pH, sensory traits, and microbiological counts) was measured in replicates (4–12 replicates depending on the trait) during the storage period (42 days). The pooled effect of treatments during the storage period on quality traits (chemical, physical, and microbiological properties) was evaluated by ANOVA. The model investigated the main effects of treatments as well as the interaction effect (between treatments and the time of storage) using the general linear model (GLM) in SPSS version 18.0 (SPSS Inc., Chicago, IL, USA) for the main quality traits of fresh oregano. The means were separated using Tukey’s range test, with *p* ≤ 0.05 considered significant.

## 3. Results

### 3.1. Chemical Compositions of Fresh Oregano

Table 1 shows the proximate chemical analysis of fresh oregano including moisture, ash, fat, and fiber constituents.

### 3.2. pH Measurement Analysis

**Figure 2:**
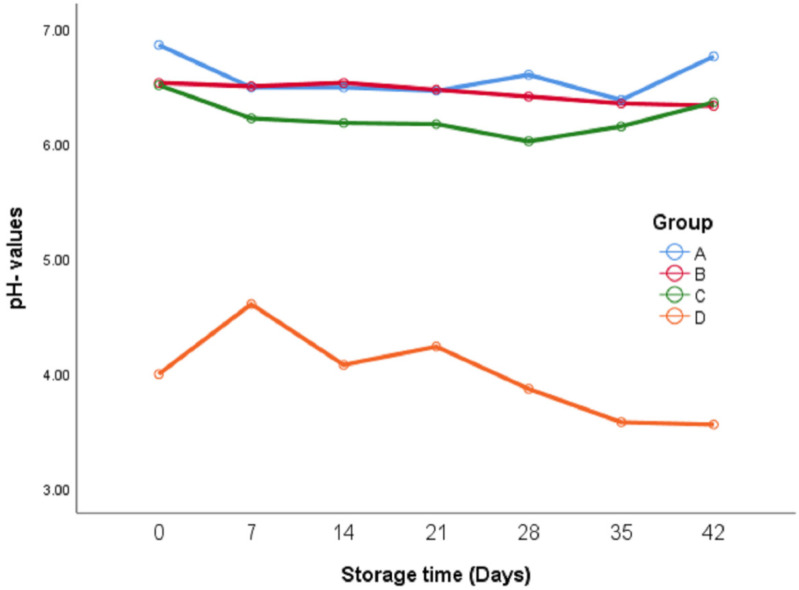
pH of fresh oregano at refrigerator temperature 4 °C during storage period (42 days). Group A: (Control group): (100% Fresh oregano leaves), Group B: (73.2% Fresh oregano, 15% Fresh *Allium cepa*, 20% corn oil, 1.8% salt), Group C: (61.9% Fresh oregano, 15% Fresh *Allium cepa*, 20% corn oil, 1.8% salt, 1.29% sumac “*Rhus coriaria*”), Group D: (59.2% Fresh oregano, 15% Fresh *Allium cepa*, 20% corn oil, 1.8% salt, 4% lactic Acid, ultimate pH 4.4.

pH was measured during the refrigerated storage period (Figure 2) to understand the effect of microbial growth on pH. The initial pH for group A was significantly higher than the other groups, which represented the natural pH of oregano leaves (there were no additives). Group D exhibited the lowest significant value of all groups. There was no significant difference between groups B and C. The addition of sumac led to a reduction of about 0.3 units of pH, while the addition of lactic acid to group D caused a reduction in pH of about 2.8 units in comparison with control group A. In general, there were no significant changes in pH during the storage period in groups A, B, and C.

### 3.3. Microbiological Analysis

**Figure 3:**
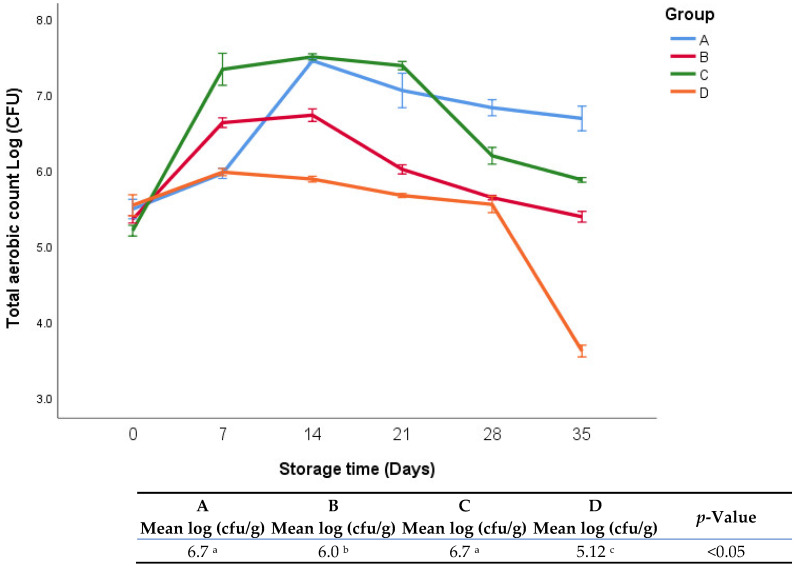
Total aerobic bacterial count of fresh oregano at refrigerator temperature 4 °C during storage period (35 days). The significant differences (*p* < 0.05) between means are indicated in different letters in the same row. The effect of treatments was pooled to consider the whole storage period and the means were separated by Duncan test.

Total plate count was used as an indicator of bacterial aerobic populations in different groups. It is not a measure of the entire bacterial population but rather a generic test for organisms that grow aerobically at mesophilic temperatures (25 to 40 °C). Figure 3 shows the growth of total bacteria during the storage period (42 days). There was an interaction between the effects of treatments and the effects of storage time. Groups A and C exhibited significantly (*p* < 0.05) higher aerobic counts than groups B and D during the entire period of storage.

### 3.4. Anaerobic Bacteria Analysis

**Figure 4:**
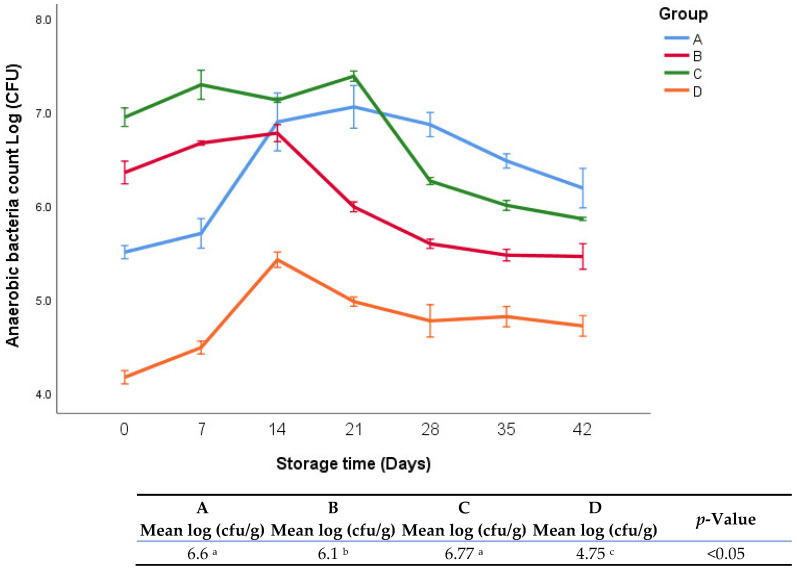
Anaerobic bacterial count of fresh oregano at refrigerator temperature 4 °C. The significant differences (*p* < 0.05) between means are indicated in different letters in the same row. The effect of treatments was pooled to consider the whole storage period and the means were separated by Duncan test.

The anaerobic bacterial count during the storage period (35 days) at refrigerator temperature is shown in Figure 4. There was a significant interaction between treatments and storage time. There was no significant difference in total anaerobic count between groups A and C during the whole storage period. Group D exhibited significantly lower anaerobic counts than the other groups. In general, the results of aerobic and anaerobic counts for all groups were quite similar. It was obvious that group D exhibited the lowest anaerobic bacteria counts during the whole period of storage.

**Figure 5:**
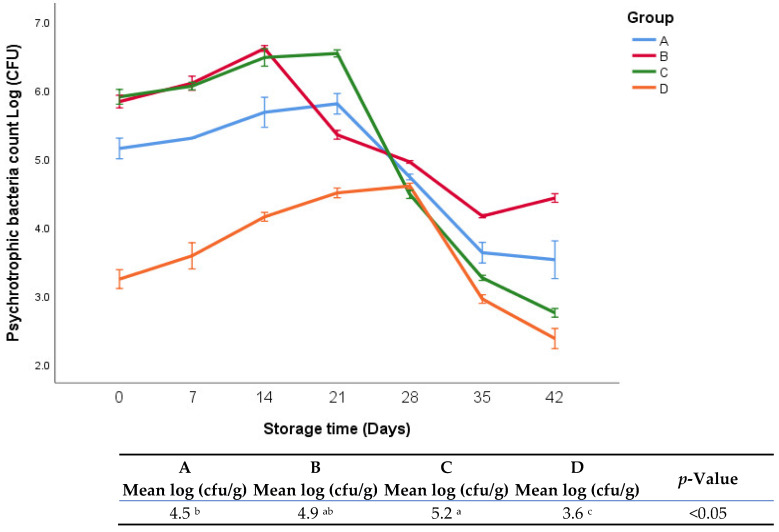
Psychrotrophic bacterial count of fresh oregano samples at refrigerator temperature 4 °C. The significant differences (*p* < 0.05) between means are indicated in different letters in the same row. The effect of treatments was pooled to consider the whole storage period and the means were separated by Duncan test.

The growth of psychrotrophic bacteria in fresh oregano leaves at refrigerator temperature 4 °C is shown in Figure 5. It was clear that group D exhibited significantly lower psychrotrophic bacterial count during the whole period of storage than the other groups. There were moderately significant differences between groups A and B (4.5 vs. 4.9, *p* < 0.05), respectively. Group C exhibited a significantly higher psychrotrophic bacterial count than group A (5.2 vs. 4.5, *p* < 0.05), respectively.

**Figure 6:**
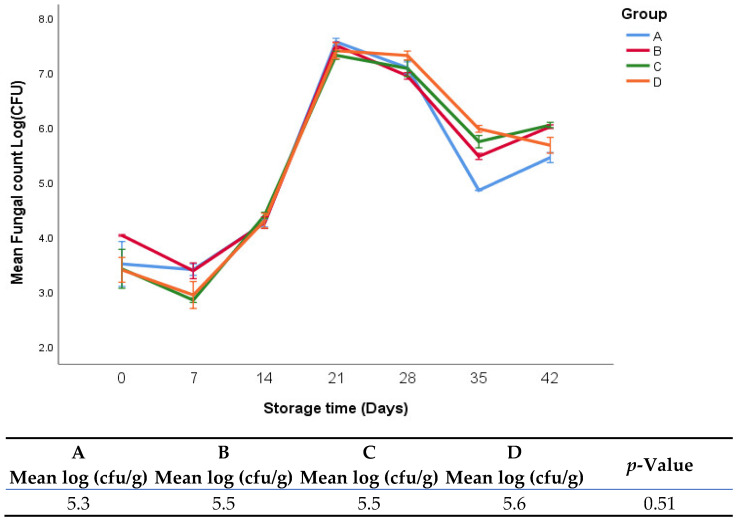
Mold and yeast growth of fresh oregano at refrigerator temperature 4 °C. The significant differences (*p* < 0.05) between means are indicated in different letters in the same row. The effect of treatments was pooled to consider the whole storage period and the means were separated by Duncan test.

The growth of yeast and mold in fresh oregano leaves at a refrigerator temperature 4 °C is shown in Figure 6. In general, there were no significant differences in yeast and mold counts during the whole period of storage for all groups.

### 3.5. Color Index (L*a*b*)

**Figure 7:**
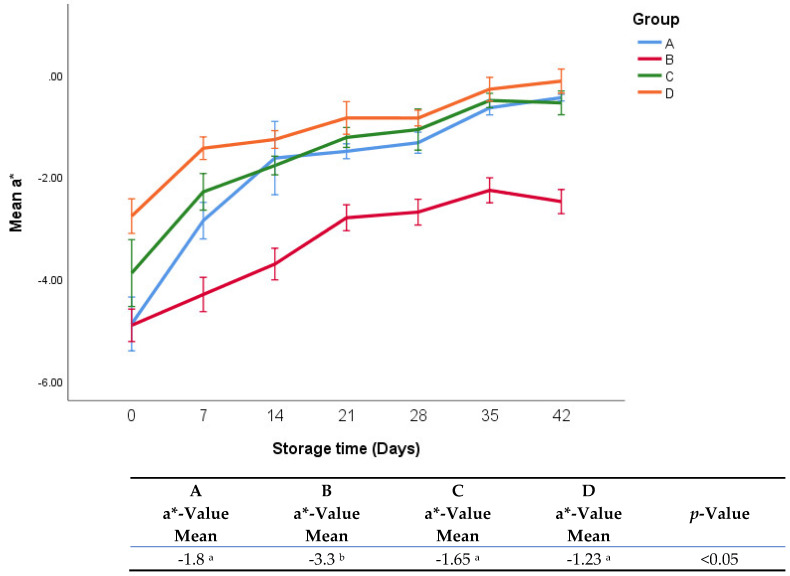
a*-values of fresh oregano at refrigerated temperature 4 °C during storage period (42 days). The significant differences (*p* < 0.05) between means are indicated in different letters in the same row. The effect of treatments was pooled to consider the whole storage period and the means were separated by Duncan test.

The effects of treatments on a*-values during storage are shown in Figure 7. There were no significant differences between groups A, C, and D. During the whole period of storage, the a*-values of Group B changed much less than those of the other groups. Overall, group B exhibited significantly lower a-values than the other groups.

**Figure 8:**
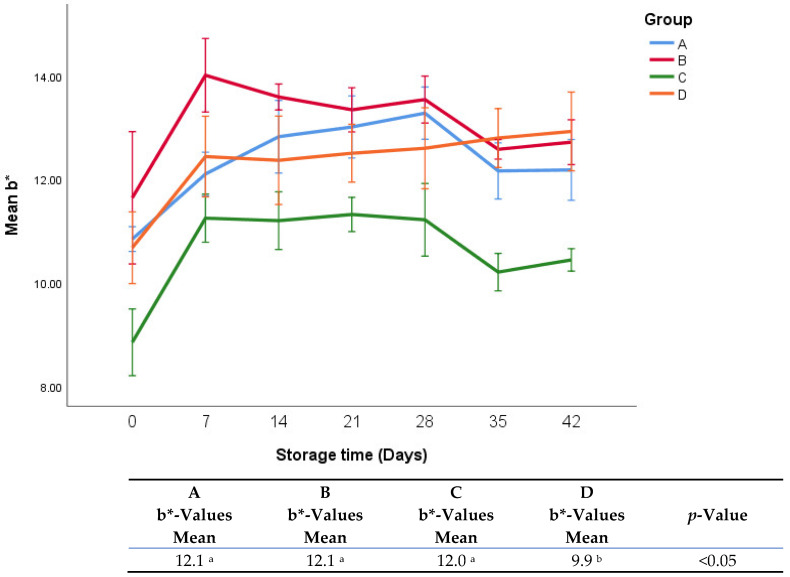
b*-values of fresh oregano at refrigerated temperature 4 °C during storage period (42 days). The significant differences (*p* < 0.05) between means are indicated in different letters in the same row. The effect of treatments was pooled to consider the whole storage period and the means were separated by Duncan test.

**Figure 9:**
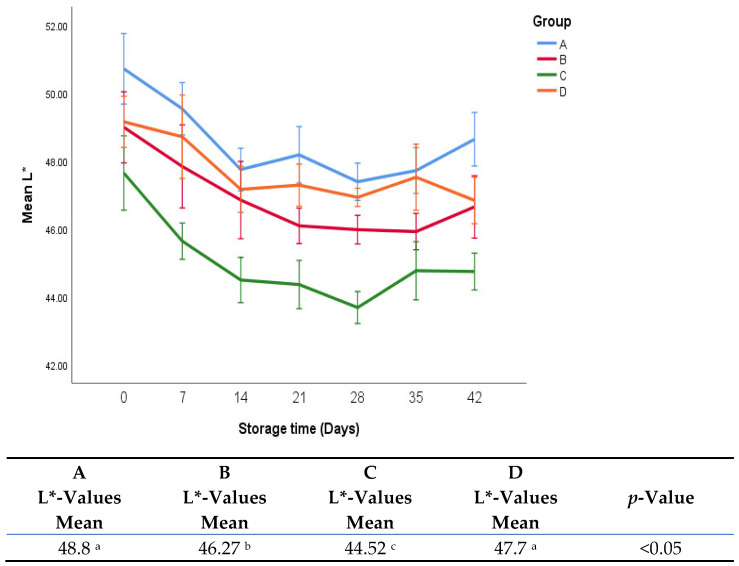
L*-values of fresh oregano at refrigerated temperature 4 °C during storage period (42 days). The significant differences (*p* < 0.05) between means are indicated in different letters in the same row. The effect of treatments was pooled to consider the whole storage period and the means were separated by Duncan test.

The effects of treatments on b*-values and L*-values are shown in Figure 8 and Figure 9. It was found that there were no significant differences in b*-values between groups A, B, and C. Group D had significantly lower b*-values (9.9 vs. 12.1, 12.1, and 12.0, *p* < 0.05) than groups A, B, and C. It was observed that there was a sharp increase in b*-values in the first week in all groups.

It was found that there were no significant differences in L*-values between group A and D. Group C exhibited significantly lower L*-values than the other groups. There was a drop in L*-values in the first two weeks of storage in all groups.

### 3.6. Sensory Analysis

**Figure 10:**
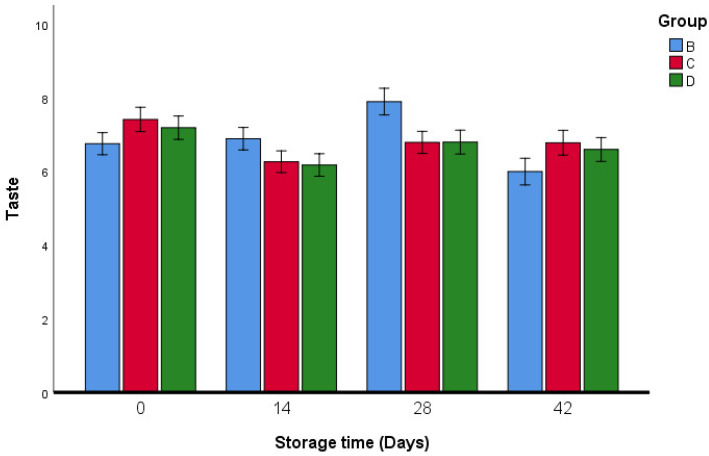
Taste analysis of fresh oregano during storage period (42 days). Group B: (73.2% Fresh oregano, 15% Fresh *Allium cepa*, 20% corn oil, 1.8% salt), Group C: (61.9% Fresh oregano, 15% Fresh *Allium cepa*, 20% corn oil, 1.8% salt, 1.29% sumac “*Rhus coriaria*”), Group D: (59.2% Fresh oregano, 15% Fresh *Allium cepa*, 20% corn oil, 1.8% salt, 4% lactic Acid, ultimate pH 4.4.

**Figure 11:**
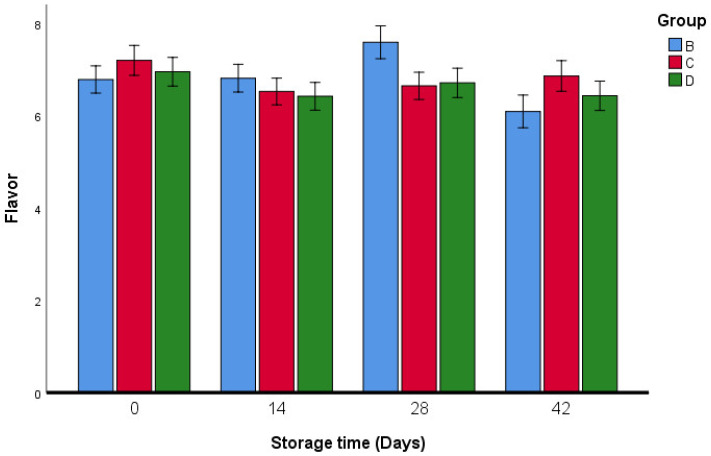
Flavor sensory analysis of fresh oregano during storage period (42 days). Group B: (73.2% Fresh oregano, 15% Fresh *Allium cepa*, 20% corn oil, 1.8% salt), Group C: (61.9% Fresh oregano, 15% Fresh *Allium cepa*, 20% corn oil, 1.8% salt, 1.29% sumac “*Rhus coriaria*”), Group D: (59.2% Fresh oregano, 15% Fresh *Allium cepa*, 20% corn oil, 1.8% salt, 4% lactic Acid, ultimate pH 4.4.

**Figure 12:**
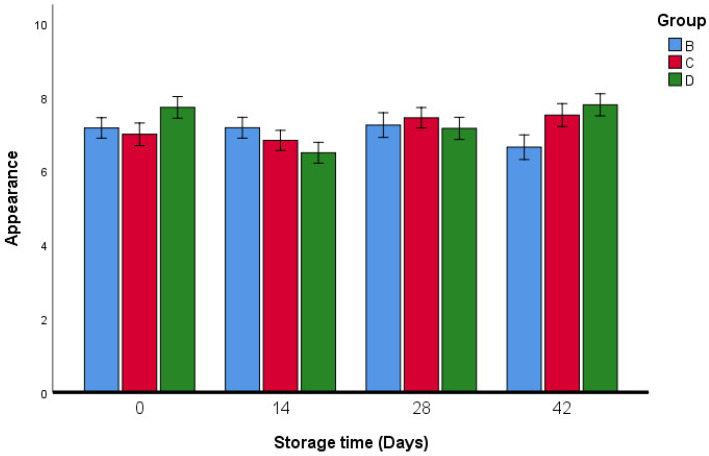
Appearance analysis of fresh oregano during storage period (42 days). Group B: (73.2% Fresh oregano, 15% Fresh *Allium cepa*, 20% corn oil, 1.8% salt), Group C: (61.9% Fresh oregano, 15% Fresh *Allium cepa*, 20% corn oil, 1.8% salt, 1.29% sumac “*Rhus coriaria*”), Group D: (59.2% Fresh oregano, 15% Fresh *Allium cepa*, 20% corn oil, 1.8% salt, 4% lactic Acid, ultimate pH 4.4.

**Figure 13:**
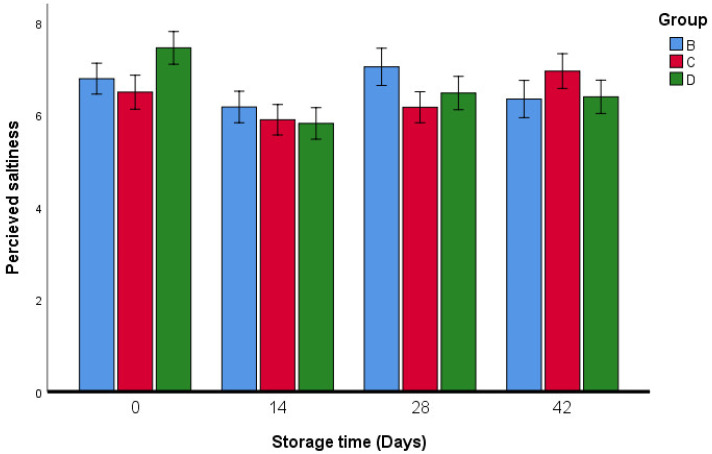
Saltiness sensory analysis of fresh oregano during storage period (42 days). Group B: (73.2% Fresh oregano, 15% Fresh *Allium cepa*, 20% corn oil, 1.8% salt), Group C: (61.9% Fresh oregano, 15% Fresh *Allium cepa*, 20% corn oil, 1.8% salt, 1.29% sumac “*Rhus coriaria*”), Group D: (59.2% Fresh oregano, 15% Fresh *Allium cepa*, 20% corn oil, 1.8% salt, 4% lactic Acid, ultimate pH 4.4.

The results of the sensory analysis of fresh oregano samples are reported in Figure 9, Figure 10, Figure 11, Figure 12 and Figure 13. Breaded oregano (*qrass za’atar*) samples were obtained using the same traditional dough as that used by the local baker. The two fresh oregano samples in groups C and D proved to have similar characteristics (taste, flavor, saltiness, and appearance) to fresh oregano leaves that were produced from the traditional bakery (group B). Our study showed that there were no significant differences in taste between groups B, C, and D, except in group B, the taste was different on the last day of the storage period (Figure 10).

However, in all of the studied recipes, there were significant differences in the flavor and appearance of the fresh oregano pastries produced using different ingredients (Figure 11 and Figure 12).

**Figure 14:**
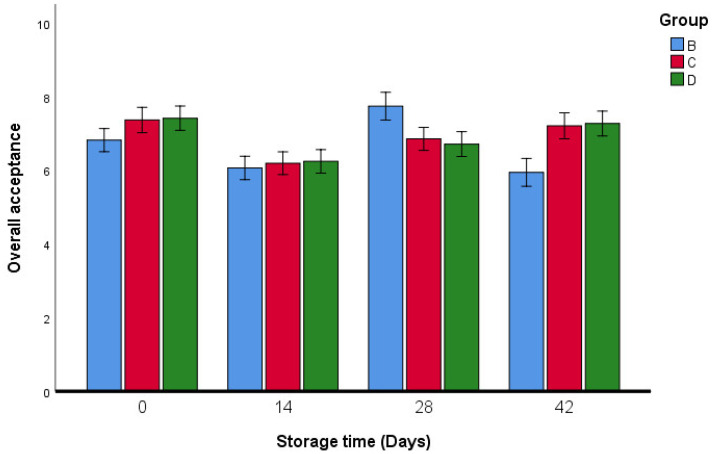
Overall acceptance sensory analysis of fresh oregano during storage period (42 days). Group B: (73.2% Fresh oregano, 15% Fresh *Allium cepa*, 20% corn oil, 1.8% salt), Group C: (61.9% Fresh oregano, 15% Fresh *Allium cepa*, 20% corn oil, 1.8% salt, 1.29% sumac “*Rhus coriaria*”), Group D: (59.2% Fresh oregano, 15% Fresh *Allium cepa*, 20% corn oil, 1.8% salt, 4% lactic Acid, ultimate pH 4.4.

All samples were judged to be satisfactory for perceived saltiness. Group D (which had lactic acid) showed significant differences in perceived saltiness only on day 42 (Figure 13). According to the evaluation of the overall acceptance (Figure 14), the oregano bread (*qrass za’atar*) produced using the different oregano mixes was the most appreciated by panelists. However, some complaints during the testing of group C were recorded by some of the tasters, who indicated a bitter taste.

## 4. Discussion

The proximate chemical compositions of local species of fresh oregano have not been well reported. The composition is usually affected by different factors such as cultivars, seasons, soil, and farming conditions [17]. This aromatic plant is characterized by a high density of nutrients such as minerals, vitamins, and phytonutrients [22]. Wide variations in the chemical compositions have the potential to contribute to the nutritional and health needs of consumers [22]. The results of proximate analysis are in agreement with previous studies conducted on thyme [17,23].

With respect to pH during the storage period, there was a significant decrease in pH in some groups, in particular group D. This may be due to the growth of lactic acid bacteria. In general, there were similarities in the patterns of changes in pH between groups stored at room conditions to those observed in a previous study [18]. The growth of lactic acid in group D also explains the gap in pH compared with the other groups. Cabello-Olmo et al. (2020) found a drop in pH in some vacuum-packed foods [24].

The pH of group A was the highest of all groups (Figure 2), which can be explained by the absence of any additives as in the control group. On the other hand, the lowest growth of aerobic bacteria was significantly observed in group D against the other groups (5.12 vs. 6.7, 6, and 6.7 logs, respectively). Group B exhibited a lower bacterial count than groups A and C; this may be attributed to the effect of onion addition. It was reported that allicin in onions had antifungal and antibacterial activity [25]. However, it is important to note that although group C contained onions, the addition of sumac may have contributed to the increase in the bacterial count. The sumac that was used in this experiment was not sterilized. The bacterial count in this study was quite lower than that in a similar previous study that was carried out at room temperature [18]. The growth of bacteria was lower at low temperatures, which meant the organism was not able to supply the maintenance requirement of the growth rate-limiting nutrient because of loss of affinity for that substrate [25].

Overall, the total anaerobic counts were relatively low across all groups (A, B, C, and D: 6.6, 6.1, 6.7, and 4.7 logs, respectively). This may be related to the combined effect of natural additives and refrigerated conditions. As mentioned earlier, the lower anaerobic count and sharp pH drop in group D could be associated with lactic acid. Our findings were in alignment with our previous study [18], which was carried out in similar experimental conditions except different storage temperature (4 vs. 25 °C). The sole difference between the current experiment and the previous one was in the absolute microbial count, where in the current study, the microbial counts were lower than the previous study, which can be attributed to differences in storage temperature. In this context, Russell et al. [26] found that low temperatures reduced the metabolic processes of microorganisms, which in turn led to a decrease in bacterial growth. In another study, it was found that cold environments disturbed homeostasis, which led to a drop in the growth rate of microorganisms [27]. Generally, psychrotrophic bacterial counts during the whole period of storage for all groups were relatively not high (the highest count was 5.2 logs in group C). This result may be attributed to the combined effect of natural additives, vacuum packaging, and cold environment, which led to improving the reduction performance in the growth of bacteria. Additionally, similar to the anaerobic bacterial growth, the slower rate in the growth of psychrotrophic bacteria in group D during the storage period may be attributed to the very low ultimate pH resulting from the addition of lactic acid.

In respect to fungal growth, our findings may be related to storage temperature, which is one of the most influential factors on yeast and mold growth [28]. Fungi can live in a relatively large range of temperatures, but growth and metabolism rates change at different temperatures even when other conditions, e.g., nutrient and water activity, are constant [29]. Yeast and mold growth were found to be lower at 10 °C compared with 15, 20, 25, and 30 °C [30].

In general, there was a gradual increase in a*-values during the whole period of storage in all groups. It was found that onion (*Allium Cepa* L.) reduced browning reactions [25], which may explain why group B had the lowest change in a*-value, while groups C and D contained sumac and lactic acid in addition to onions, which may have counteracted the effect of the onion.

The increase in b*-values in the first week of storage can be attributed to the degradation of chlorophylls and carotenoids during storage due to oxidative reactions of phenolic compounds by polyphenol oxidase, which produces quinones to various polymerized products [31,32]. The degradation of chlorophyll is usually very high at low pH, which may explain why group D exhibited the lowest b*-value [32].

L*-values in group C were significantly higher than in the other groups. This can be attributed to the addition of sumac, which contains a high level of natural pigments that may have contributed to darkening the color of oregano leaves.

There were no significant differences in overall sensory traits between groups, but perceived saltiness was higher in groups C and D, which may have been due to the addition of sumac and lactic acid.

## 5. Conclusions

Our study showed that it is possible to formulate fresh oregano recipes that are stable for a reasonable storage period. The most efficient addition for preserving various quality characteristics during storage was lactic acid. It was discovered that the stability of the oregano blend was much enhanced by the cold storage. Due to its limited storage stability, this product has not yet been fully utilized in the export market. Nevertheless, the results of this study may help to increase storage stability for such products.

## Figures and Tables

**Table 1:** Proximate chemical composition of fresh oregano leaves.

	Composition	Mean ± SD (g/100 g)
1	Moisture	66.9 ± 0.42
2	Fiber	20.51 ± 1.32
3	Proteins	0.71 ± 0.05
4	Ash	5.50 ± 0.22
5	Fat	6.33 ± 0.22

## Data Availability

Data is contained within the article.
